# UPLC-ESI-QTOF-MS^2^-Based Identification and Antioxidant Activity Assessment of Phenolic Compounds from Red Corn Cob (*Zea mays* L.)

**DOI:** 10.3390/molecules23061425

**Published:** 2018-06-12

**Authors:** María Hernández, Janeth Ventura, Cecilia Castro, Víctor Boone, Romeo Rojas, Juan Ascacio-Valdés, Guillermo Martínez-Ávila

**Affiliations:** 1 Department of Food Science and Technology, School of Chemistry, Autonomous University of Coahuila, Saltillo, Coahuila 25280, Mexico; hernandez_m@uadec.edu.mx (M.H.); alberto_ascaciovaldes@uadec.edu.mx (J.A.-V.); 2 School of Health Science, Autonomous University of Coahuila, Piedras Negras, Coahuila 26090, Mexico; janethventura@uadec.edu.mx; 3 Laboratory of Chemistry and Biochemistry, School of Agronomy, Autonomous University of Nuevo León, General Escobedo, Nuevo León 66050, Mexico; caslopcec28@hotmail.com (C.C.); romeo.rojasmln@uanl.edu.mx (R.R.); 4 School of Medicine, Autonomous University of Coahuila, Piedras Negras, Coahuila 26090, Mexico; victor_boone@my.uvm.edu.mx

**Keywords:** phenols compounds, red corn cob, ultrasonic-assisted extraction, UPLC-MS^2^

## Abstract

In this study, the extraction of phenolic antioxidants from red corn cob was carried out using ultrasound-assisted extraction (UAE). The solid:liquid ratio and extraction time were evaluated when obtaining these bioactive compounds. The total phenolic contents were evaluated using the Folin Ciocalteu method, while the antioxidant activity was measured by ABTS^•+^ and DPPH^•^ assays. The amount of phenolic compounds ranged from 215.17 ± 33.49 to 527.33 ± 103.79 GAE mg/100 g and, overall, high solid:liquid ratios and time periods release more phenolic compounds. Moreover, the red corn cob extracts showed higher radical scavenging capacity according to the results obtained using the ABTS^•+^ technique compared to the DPPH^•^ test. The coupling of liquid chromatography and mass spectrometry assay allowed the determination of 11 phenolic compounds, including phenolic acids and flavonoids. Thus, our results demonstrated for the first time the potential of red corn cob as a source of bioactive compounds, which might be included in food and pharmacological preparations.

## 1. Introduction

Mexico has a wide variety of pigmented corns (*Zea mays*) with different colors, such as yellow, orange, red, blue, purple, and black. The presence of pigments is attributed to a high content of flavonoid-type phenolic compounds [1]. Nowadays, there has been increased interest in these bioactive compounds, which is mainly related to their potential use in pharmacy and food industries. This is due to their diverse biological activities, such as anticancer, antioxidant, anti-microbial, cardioprotective, and other activities [2,3]. Phenolic compounds can act as antioxidants since they are able to scavenge the free radicals that donate hydrogen atoms or electrons. Furthermore, they are able to chelate metallic ions with these properties related to its chemical structure [4,5]. As reported in the scientific literature, the phenolic compounds in corn can include phenolic acids, such as ferulic, *p*-coumaric, and vanillic acids their derivatives; flavonoids, such as quercetin and hesperetin; and different types of anthocyanins [3,6,7]. However, the extraction of these compounds from plant materials, including corn cobs, requires processing for a long time, which may cause degradation and loss in the antioxidant activity. Ultrasound-assisted extraction (UAE) is a technique that provides an efficient extraction process in a short period of time. This is based on the phenomenon of cavitation generated by the propagation of the ultrasonic pressure waves combined with localized temperatures, which causes the breakage of the cell wall and the liberation of the cell contents to the medium [8]. UAE has been successfully used to obtain phytochemicals with different biological activities from cellulosic plant materials, such as the stems and roots of *Jatropha dioica* and *Turneradiffusa* leaves [9]. Thus, this technique could offer great advantages for obtaining the bioactive compounds from corn cobs. To the best of our knowledge, there have been no reports about the extraction and identification of bioactive compounds from red corn cob. In this sense, the aim of the present work was to carry out the extraction and identification of phenolic compounds in these byproducts using a highly sensitive analytical technique, which was, namely, ultra-performance liquid chromatography coupled to electrospray ionization quadrupole-time of flight mass spectrometry (UPLC-ESI-QTOF-MS^2^).

## 2. Results and Discussion

### 2.1. Phenolics Extraction by UAE

The extraction of phenolic compounds from different parts of maize has been carried out using organic solvents (different to ethanol) and chemical solutions [10], which could considerably increase the pollution in the environment and limit their use in food applications. In this sense, it has recently been reported that the use of GRAS (generally recognized as safe) solvents, including water, ethanol, and their mixtures, offer great opportunities for obtaining high-quality and high-phenolic content extracts from different plant materials, including the byproducts derived from maize [11,12].

The amount of phenolic compounds found in this study ranged from 215.17 ± 33.49 to 527.33 ± 103.79 GAE mg/100 g and, overall, high solid:liquid ratios and time periods release more phenolic compounds. The obtained results were comparable to those obtained by Lopez-Martinez et al. [13], who reported that the amount of phenolics was in the range of 283–617 GAE mg/100 g in red-colored Mexican maize flour, suggesting that red corn cob is a byproduct with important levels of bioactive compounds compared to other food materials that are related to the food industry. However, our values are lower than those previously recorded by Egüés et al. [14] and Mazewski et al. [3], who evaluated corn stalks and whole kernels from red corn as sources of phenolic compounds. This suggests that the level of these compounds depends on the cultivars and species. Furthermore, the extracting factors have an influence on the recovery of bioactive molecules from these plant materials. The values of total phenolic compounds are shown in Table 1 for all treatments. It was observed that the solid:liquid ratio and extraction time affected the yield of phenolics, which significantly increased when higher values of these variables are used for extraction. Our findings are consistent with those previously reported by Monroy et al. [11], who reported an increased extraction of phenolic compounds when the solid:liquid ratio (crushed purple corn cob + co-solvent ethanol:water) was increased. In addition, due to the polarity of the phenolics, a greater recovery is expected with the addition of polar solvents [15]. Moreover, it has been reported that the antifungal properties of phenolic compounds from other lignocellulosic materials obtained with a hydro-ethanolic (50:50) solvent are more effective antimicrobial agents compared to hydro-methanolic extracts [16]. In this sense, the use of ethanol and water mixtures in the recovery of bioactive compounds from red corn cob could be considered as a good alternative since ethanol is a low-cost, readily available, and less environmentally dangerous solvent compared with other extracting agents. Furthermore, to the best of our knowledge, this is the first scientific study that has indicated that UAE can be used for the recuperation of phenolic compounds from red corn cobs. This technique has been successfully used in the extraction of phenolic antioxidants from other cellulosic plant materials, which requires aggressive conditions to disrupt their structure in order to improve the contact of the solvent with the cell wall and to generate the ultrasonic cavitation phenomenon [17]. Thus, the use of GRAS solvents and emerging technologies as UAE allow us to obtain bioactive compounds with antioxidant activity, as described below. 

### 2.2. Antioxidant Activity of Red Corn Cob Extracts 

Antioxidant activity describes the ability of redox molecules in foods and biological systems to scavenge free radicals [18]. In this study, two assays based on single electron transfer were performed in order to estimate this activity in red corn cob extracts. The extracts showed a slightly higher radical scavenging capacity according to the results obtained using the ABTS^•+^ technique compared to the results obtained using DPPH^•^ (Table 1). This pattern is similar to the pattern observed early by Lopez-Martinez et al. [13], who determined the antioxidant capacity of kernels of eighteen strains of Mexican maize. According to Floegel et al. [18], higher antioxidant capacities were recorded by the ABTS^•+^ assay compared to the DPPH^•^ test in highly pigmented food samples. Therefore, the differences between antioxidant activities observed in this study could be explained due to the flavonoid-type compounds being mostly responsible for most blue, red, and other colors in plant materials [19]. Thus, these types of phenolics are the most important bioactive compounds identified in the red corn cob extract. Since a high correlation was described between the level of phenolic compounds and antioxidant activity measured by ABTS^•+^ and DPPH^•^ assays in several food samples and wastes [18], these compounds can be considered as the major contributor to the antioxidant capacity of red corn cobs. 

### 2.3. Identification of Compounds by UPLC-ESI-QTOF-MS^2^

**Figure 1:**
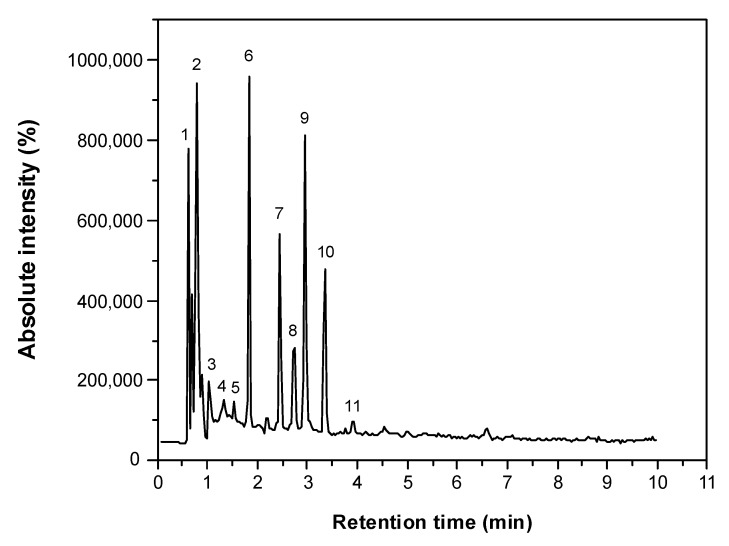
Chromatogram of red corn cob extract UAE sample using UPLC-Q/TOF-MS^2^ in negative ionization mode. For signal assignation, see Table 2.

**Figure 2:**
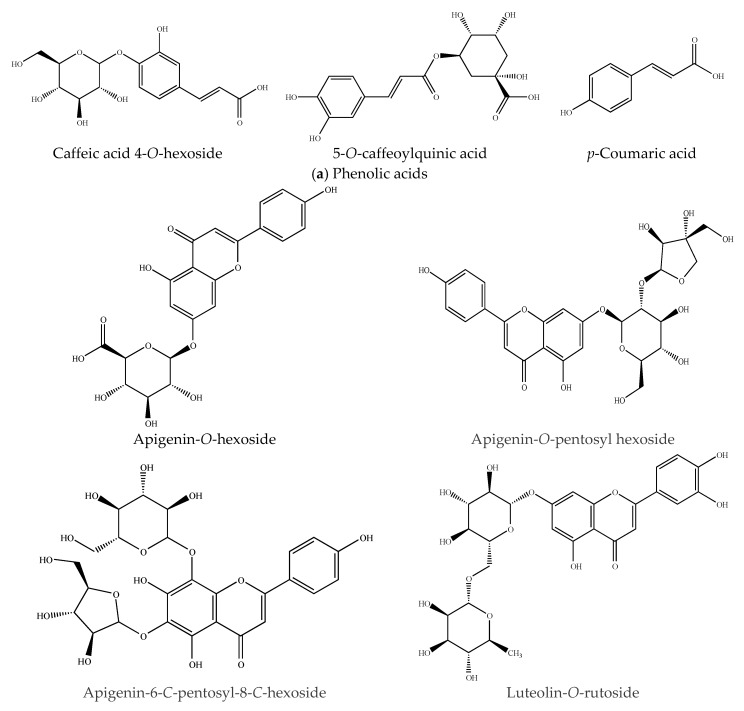

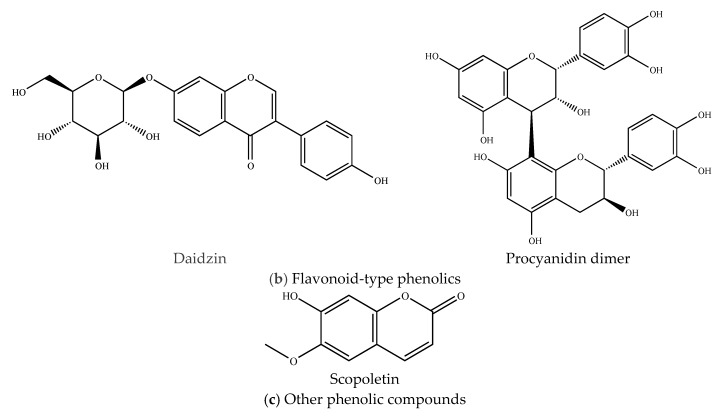
Phenolic compounds identified in red corn cob extract.

The application of mass spectroscopy has been successfully applied in the identification of phenolic compounds from several cellulosic plant materials [20,21,22]. In this study, we used the UPLC-ESI-QTOF-MS^2^ technique, which allows the identification of chemical molecules by interpreting their MS and MS/MS spectra determined by QTOF-MS and comparing them to scientific literature and open-access mass-spectra databases. The phenolic profiles and chromatograms obtained from the UAE technique are summarized in Table 2 and Figure 1, respectively. The signals detected are numbered by their elution order (retention time). Eleven peaks in total were registered in the red corn cob extract, which mainly belong to two principal metabolite types: phenolic acids and flavonoids. The compounds were eluted in a very short period of time (6 min). The short period of time and number of peaks indicates that the UPLC is a good technique for separating these bioactive compounds. The polyphenolic analysis of this plant material allowed for the identification of at least 10 compounds (Figure 2), which can be used for potential applications in the food industry.

#### 2.3.1. Phenolic Acids

Peaks 1, 4 and 10 were recorded as three hydroxycinnamic acids (Table 2). Peak 1 was detected at RT = 0.78 and [M − H]^−^ = 387.0324, which was tentatively assigned to caffeic acid 4-*O*-hexoside based on its MS spectrum with a fragment ion at *m*/*z* 135.0156 ion that is consistent with deprotonated caffeic acid. The 5-*O*-caffeoylquinic acid, with a [M − H]^−^ ion at *m*/*z* 353.0301, was the proposed compound represented by peak 4 due to its dominant fragment ion (*m*/*z* 191.0182), which corresponds to deprotonated quinic acid [12]. Peak 10 (RT = 3.35 and *m*/*z* = 163.0404) was identified as *p*-coumaric acid, since its major fragments were found at [M − H]^−^ = 119.0502 (loss of a formic acid moiety) and [C_6_H_3_O]^−^ ions at *m*/*z* 93.1266, which is consistent with previously reported data by Barnaba et al. [5], this compound has previously been identified in corn stalks [23].

#### 2.3.2. Flavonoid-Type Phenolics

Four glycosides were identified in the extract of red corn cob, three of which correspond to the apigenin derivatives that were assigned to Peaks 3, 7 and 8, respectively. The first one was assigned to apigenin-*O*-hexoside, which had a [M − H]^−^ ion at *m*/*z* 445.0452 and a fragment at *m*/*z* 269.0238 corresponding to apigenin moiety. The second one was assigned to apigenin-*O*-pentosyl hexoside, which had a deprotonated [M − H]^−^ ion at *m*/*z* 563.0032 with a fragmentation pathway (*m*/*z* 563, 269.0126) reflecting the presence of pentosyl (1→2) hexoside. Finally, the third one was denoted as apigenin 6-*C*-*pentosyl*-8-*C*-hexoside based on its molecular ion [M − H]^−^ at *m*/*z* 563.0036, which released two MS^2^ fragment ions at *m*/*z* 563, and 473 that correspond to loss of water [M − H]^−^ and cross-ring cleavage of the sugar unit, which is [M−90]^−^ for C-pentosides. These compounds were consistent with a previous report by Piasecka et al. [24], Šulniūtė et al. [25] and Farag et al. [26]. Additionality, the peak 5 with RT = 1.83 exhibited a base peak [M − H]^−^ at *m*/*z* 593.0031 and was assigned as luteolin-*O*-rutinoside since it showed a common MS^2^ base peak ion at *m*/*z* 285.0391 of the aglycone, that corresponded to the loss of a rutinose moiety (308 Da) [27]. Finally, peak 6 (RT = 2.16), which was tentatively identified as daidzin, had a deprotonated [M − H]^−^ ion at *m*/*z* 415.0813 with a predominant fragment ion at *m*/*z* 253.0213. This is consistent with the loss of hexose [M – H − 162]^−^, thus releasing the aglycone daidzein [28]. In addition, a condensed tannin was identified as a procyanidin dimer, which was eluded at RT = 3.89 and contained [M − H]^−^ = 577.0161 with a dominant fragment ion at *m*/*z* = 289.0143, which matches with the loss of a catechin unit [12].

#### 2.3.3. Other Phenolic Compounds

According to the chromatogram of bioactive compounds (Figure 1), one of the most abundant phenolic compounds present in the red corn cob extract (peak 2, RT = 0.88) was tentatively identified as scopoletin, which is an hydroxycoumarin with a molecular ion at [M − H]^−^ = 191.0487. This has a lost methyl group in the negative mode that follows the ester group, which was confirmed by the fragment ion at *m*/*z* 176.0112 [5,28]. Finally, this analysis also revealed a peak (RT = 2.97) at [M − H]^−^ = 237.0558, which corresponded to another non-identified phenolic compound. This unknown compound may contribute to the biological activities of the corn cob together with the identified phenolic antioxidant. Most of the studies in the literature have focused on purple varieties of corn but, to our knowledge, there are no published reports with red corn cobs as a source of phenolics.

## 3. Materials and Methods

### 3.1. Chemicals and Reagents

Folin Ciocalteu reagent, gallic acid, ABTS^●+^ (2,2′-azino-bis-(3-ethylbenzotiazoline-6-sulphonic acid), Trolox (6-hydroxy-2,5,7,8-tetramethylchroman-2-carboxylic acid), DPPH^•^ (2,2-diphenyl-1-picrylhydrazyl), and ethanol were purchased from Sigma Aldrich (Toluca, México). Ammonium persulfate (K_2_S_2_O_8_) and sodium carbonate (Na_2_CO_3_) were of analytical grade. Acetonitrile, methanol, formic acid, and water were of LC-MS grade and were purchased from Fisher Scientific Chemicals (Fair Lawn, NJ, EE.UU.). 

### 3.2. Plant Material

In November–December 2015, red maize was collected from local farmers in Guanajuato, México. After corn husking, the kernel cob was obtained, dried in a sunny area for two days at an ambient temperature (natural post-harvest drying), and pulverized using a ball grinder (Pulvex, Mexico). Finally, the obtained powder was stored in black containers at room temperature until analysis was conducted. 

### 3.3. Extraction of Phenolic Compounds

The extraction of phenolic compounds was performed according to the ultrasonic-assisted extraction method that was previously reported by Mane et al. [30] with slight modifications. Briefly, ethanol (55%) was employed as the extracting solvent, while three solid:liquid ratios (1:10, 1:20, and 1:30 *w*/*v*) and three different extraction times (60, 90, and 120 min) were evaluated. All the extractions were carried out at room temperature (25 °C). A BRANSON 2510 ultrasonic water bath (Marshall Scientific, NH, USA) at 40 kHz with 100% potency was used for the extraction. The obtained extracts were centrifuged at 10,000 rpm for 10 min at 4 °C and frozen at −20 °C.

### 3.4. Phenolic Content Analysis

The Folin Ciocalteu method was adapted for the quantification of the total phenolic compounds (TPC) [31]. Briefly, 25 µL of extract was mixed with 25 µL of the Folin Ciocalteu reagent, before 25 µL of sodium carbonate (75 g L^−1^) was added to the mixture. The samples were subsequently incubated at 40 °C for 30 min in a water bath. After this, 200 µL of distilled water was added to the sample and the absorbance at 750 nm was determined using a microplate analyzer (Synergy HT Multi-Detection Microplate Reader, BioTek Instruments, Inc., Winooski, VT, USA). A reference curve was obtained using gallic acid as the standard. The TPC of the extracts were reported as mg of equivalents of gallic acid per 100 g of plant (GAE mg/100 g).

### 3.5. Antioxidant Activity Assays

Two assays based on single electron transfer were performed in order to estimate the antioxidant activity of the red corn cob extracts.

#### 3.5.1. DPPH^•^ Free Radical Scavenging

The antioxidant effect of the extracts on the DPPH^•^ radical was evaluated using the methodology proposed by Brand-Williams et al. [32] with slight modifications. A total of 5 µL of sample was mixed with 295 µL of a 60 mM DPPH^•^ solution (in methanol), before the mixture was homogenized and left in the dark for 30 min. After this period, the absorbance was recorded at 517 nm using a microplate analyzer (Synergy HT Multi-Detection Microplate Reader, BioTek Instruments, Inc., Winooski, VT, USA). The DPPH^•^ radical scavenging power was expressed as mg of gallic acid equivalents per 100 g of plant (GAE mg/100 g) according to a reference curve of this standard. 

#### 3.5.2. ABTS^•+^ Free Radical Scavenging

The antioxidant effect of the extracts on the ABTS^•+^ radical was evaluated using the methodology proposed by Van den Berg et al. [33] with slight modifications. Briefly, the anionic radical ABTS^•+^ was generated by the oxidation of ABTS with potassium persulfate. An aqueous solution of potassium persulfate (2.45 mM) was mixed with ABTS^•+^ (7 mM) with a ratio of 1:2, before the mixture was left to rest for 12 h in the dark at room temperature. After this, the ABTS^•+^ radicals were diluted with absolute ethanol and adjusted to an absorbance of 0.700 ± 0.002. For the analysis, 5 µL of extract was added to 95 µL of the ABTS^•+^ solution and homogenized. The absorbance was measured at 734 nm after 1 min in a microplate analyzer (Synergy HT Multi-Detection Microplate Reader, BioTek Instruments, Inc., Winooski, VT, USA). A reference curve was obtained using Trolox as the standard and the ABTS^•+^ radical scavenging power was expressed as mg of Trolox equivalents per 100 g of plant (TE mg/100 g).

### 3.6. UPLC-ESI-QTOF-MS^2^ Assay

For this analysis, the extract with the highest antioxidant activity (extraction time = 60 min and solid:liquid ratio = 30 *w*/*v*) was used in order to identify the phytomolecules involved in antioxidation. The identification of phenolic compounds obtained from the cobs was performed in a Waters Acquity UPLC system (Waters, Millford, MA, USA) with a BEH PHENYL (2.1 mm × 100 mm, 1.7 μL) analytic column (constant temperature of 40 °C). The compounds were separated according to the methodology reported by Kumari et al. [34] with a gradient elution and constant flow of 0.3 mL/min. The mobile phase was composed of water (with formic acid at 0.1% *v*/*v*) as phase A and acetonitrile (100%) as phase B. The gradient used was 97% of solvent A for 1.10 min, before the proportion of solvent B was changed from 5 to 15% from minute 1.10 to minute 4.40. After this, 15% of B was used for 4.60 min, before being returned to initial conditions for 1 min. The UPLC system was coupled to a mass Q-TOF orthogonal accelerated spectrometer (Q-TOF ™, WATERS, UK), which was equipped with an electrospray ionization source. The detection of the mass spectrum was completed in 10 min in the negative ion mode, which had a mass range of 50*–*1200 *m*/*z*. The optimal values for the source parameters were: capillary voltage of −3.5 y + 4.0 kV, dry gas temperature of 210 °C, gas flow of 8.0 L min^−1^, nebulizer pressure of 2 bar and a spectrum velocity of 1 Hz. The automatized MS/MS (MS^2^) assays were made using an energy of slope collision of 15–35 V with argon as the collision gas and adjustment of the exploration time every 1 s. For the identification of the bioactive compounds in all experimental samples, the obtained whole mass spectrum and the patterns of fragmentation were used. The comparison of mass spectra with the database was the main tool used for identification of the compounds.

### 3.7. Experimental Design and Statistical Analysis

The experimental design and the statistical analysis of the obtained data were performed with Minitab 17 Statistical Software (Minitab, Inc., State College, PA, USA). The experimental data were analyzed by analysis of variance (ANOVA), followed by the Tukey test to detect significant differences between the means of each factor at a significance level of *p* < 0.05. All the assays were carried out in triplicate and the data are reported as the mean value ± standard deviation. 

## 4. Conclusions

The extraction of phytochemicals from agro-industrial byproducts have attracted considerable interest from the scientific community due to their potential use in the food and pharmaceutical industries as antioxidant agents. The data obtained demonstrated that the solid:liquid ratio and extraction time had a significant impact on the recovery of phenolic compounds from red corn cob when UAE was used for this purpose. The highest antioxidant activity was found using an extraction time of 60 min and solid:liquid ratio of 30 (*w*/*v*). The polyphenolic analysis of this plant material allowed for the identification of at least 10 compounds, which can be used for potential applications in the food industry. However, in vitro and/or in vivo evaluations must be conducted in order to assess the behavior of red corn cob extracts in biological systems. 

## Figures and Tables

**Table 1:** Phenolic content and antioxidant activity of red corn cob extracts obtained by UAE.

Independent Variable		Antioxidant Activity		Total Phenolic Content
Extraction Time (min)	Solid:Liquid Ratio (*w*/*v*)	ABTS^•+^ (TE mg/100g)	DPPH^•^ (GAE mg/100 g)	F. Ciocalteau (GAE mg/100 g)
60	10	148.35 ± 8.70 ^a,b,c^	38.96 ± 7.82 ^c^	215.17 ± 33.49 ^c^
60	20	155.14 ± 3.02 ^a^	44.02 ± 1.33 ^b,c^	441.95 ± 31.82 ^a,b^
60	30	151.41 ± 2.50 ^a,b^	65.20 ± 2.25 ^a^	348.39 ± 5.02 ^b,c^
90	10	133.60 ± 3.38 ^c,d,e^	34.29 ± 3.51 ^c^	385.5 ± 49.50 ^a,b^
90	20	127.56 ± 6.96 ^d,e^	50.93 ± 7.38 ^a,b,c^	242.44 ± 3.09 ^b,c^
90	30	139.44 ± 3.51 ^b,c,d^	56.82 ± 5.54 ^a,b^	453.63 ± 6.19 ^a,b^
120	10	114.63 ± 1.31 ^e^	14.39 ± 1.20 ^d^	388.00 ± 12.03 ^a,b^
120	20	121.05 ± 4.72 ^e^	35.10 ± 5.38 ^c^	474.15 ± 4.51 ^a,b^
120	30	123.73 ± 6.42 ^e^	30.03 ± 4.19 ^c,d^	527.33 ± 103.79 ^a^

The values represent the mean ± the standard deviation of three independent measurements. Data with additional letters in superscript were statistically different (*p* < 0.05).

**Table 2:** Compounds identified by UPLC-Q/TOF-MS^2^ in red corn cob extracts obtained by UAE.

N° of Signal	R_T_ (min)	[M − H]^−^ (*m*/*z*)	Tentative Identity	Phenol Type	Molecular Formula	Dominant Fragment Ion (MS^2^)	Reference
1	0.78	387.0324	Caffeic acid 4-*O*-hexoside	Phenolic acid	C_15_H_18_O_9_	135.0156	[25]
2	0.88	191.0487	Scopoletin	Hydroxycoumarin	C_10_H_8_O_4_	176.0112	[5]
3	1.32	445.0452	Apigenin-*O*-hexoside	Flavonoid	C_21_H_18_O_11_	269.0238	[25]
4	1.52	353.0301	5-*O*-caffeoylquinic acid	Phenolic acid	C_16_H_18_O_9_	191.0182	[29]
5	1.83	593.0031	Luteolin-*O*-rutinoside	Flavonoid	C_27_H_30_O_15_	285.0391	[27]
6	2.16	415.0813	Daidzin	Isoflavonoid	C_15_H_10_O_4_	253.0213	[28]
7	2.44	563.0032	Apigenin-*O*-pentosyl hexoside	Flavonoid	C_26_H_28_O_14_	563, 269.0126	[24]
8	2.74	563.0036	Apigenin 6-*C*-pentosyl-8-*C*-hexoside	Flavonoid	C_26_H_28_O_14_	563, 473	[24]
9	2.94	237.0558	Unknown	-	-	-	-
10	3.35	163.0404	*p*-Coumaric acid	Phenolic acid	C_9_H_8_O_3_	119.0502, 93.1266	[5]
11	3.89	577.0161	Procyanidin dimer	Flavonoid	-	289.0143	[28]
